# Phytohormones Regulate the Development of Arbuscular Mycorrhizal Symbiosis

**DOI:** 10.3390/ijms19103146

**Published:** 2018-10-12

**Authors:** Dehua Liao, Shuangshuang Wang, Miaomiao Cui, Jinhui Liu, Aiqun Chen, Guohua Xu

**Affiliations:** 1 State Key Laboratory of Crop Genetics and Germplasm Enhancement, College of Resources and Environmental Sciences, Nanjing Agricultural University, Nanjing 210095, China; liao.d.h@163.com (D.L.); 13182996886@163.com (S.W.); 15850598876@126.com (M.C.); miaomiaocui0126@126.com (J.L.); ghxu@njau.edu.cn (G.X.); 2 MOA Key Laboratory of Plant Nutrition and Fertilization in Lower-Middle Reaches of the Yangtze River, Nanjing Agricultural University, Nanjing 210095, China

**Keywords:** phytohormone, signaling, DELLA, arbuscular mycorrhizal symbiosis

## Abstract

Most terrestrial plants are able to form a root symbiosis with arbuscular mycorrhizal (AM) fungi for enhancing the assimilation of mineral nutrients. AM fungi are obligate symbionts that depend on host plants as their sole carbon source. Development of an AM association requires a continuous signal exchange between the two symbionts, which triggers coordinated differentiation of both partners, to enable their interaction within the root cells. The control of the AM symbiosis involves a finely-tuned process, and an increasing number of studies have pointed to a pivotal role of several phytohormones, such as strigolactones (SLs), gibberellic acids (GAs), and auxin, in the modulation of AM symbiosis, through the early recognition of events up to the final arbuscular formation. SLs are involved in the presymbiotic growth of the fungus, while auxin is required for both the early steps of fungal growth and the differentiation of arbuscules. GAs modulate arbuscule formation in a dose-dependent manner, via DELLA proteins, a group of GRAS transcription factors that negatively control the GA signaling. Here, we summarize the recent findings on the roles of these plant hormones in AM symbiosis, and also explore the current understanding of how the DELLA proteins act as central regulators to coordinate plant hormone signaling, to regulate the AM symbiosis.

## 1. Introduction

In natural ecosystems, many plants are able to grow in nutrient-poor soils by living together with microbes, for mutual benefit, an instance which is referred to as ‘symbiosis’. Arbuscular mycorrhiza that forms by interactions between soil fungi, belonging to Glomeromycotina, and more than 80% of land plants, including the most important economic crops, such as rice, soybean, and potato, is considered to be one of the most widespread and important symbiotic associations [1]. It has been recently revealed that some presumed non-host plants, such as the model plant *Arabidopsis thaliana*, can also be colonized by the AM fungi to form rudimentary AM (RAM) phenotypes [2]. Through the AM symbiosis, AM fungi increase the nutrient availability, in particular, P and also to a lesser extent, N, S, K, and other mineral nutrients to plants; in return, they receive up to 20% of the plant-fixed carbon, for their proliferation [3]. AM interactions can also provide additional non-nutritional benefits for the hosts, such as stress tolerance and disease resistance, besides the bidirectional nutrient exchange [1]. Evidence from fossilized early land plants implies that AM association is a very ancient symbiotic system that evolved from the time when plants first colonized terrestrial environments. It is thus suggested that AM symbiosis may have offered continued advantages, for both partners, to increase fitness to adapt diverse environmental challenges [4].

Even though AM associations occur in all major land plant lineages, including many early diverging plants from hornworts, liverworts, lycopods, and ferns, only two principal morphological types, the Paris and Arum types, have been determined [5,6,7]. To establish symbiosis, AM fungi colonize the root interior, which involves morphological changes of both plant cells and fungal hyphae. Successful symbiotic interaction is preceded by mutual recognition through diffusible molecules, released by plant roots and germinating fungal spores, which triggers pre-symbiosis responses for both symbionts, to enable their interaction. Following the pre-symbiosis communication, AM fungi form entry points, such as appressoria and hyphopodia, on the host root surface, or entry coils (EC) in the epidermal or hypodermal cells, and subsequently penetrate the root and form the intraradical mycelium. In some cases, hyphae can enter the root between the two epidermal cells, or get penetration through the root hairs [8]. In the *Arum* type, hyphae spread intercellularly within the root cortex and form fine, branched tree-like structures, called arbuscules, within the cells. In the *Paris* type, thick, coiled-hyphae grow intracellularly, occasionally forming highly-branched arbuscule-like structures. Intermediate types of AM colonization combine features of both types [8,9]. In response to the invasion, the cortical cells develop a specialized membrane, termed periarbuscular membrane (PAM), to surround the branching hyphae and separate the fungus from the plant cell cytoplasm. The interfacial apoplastic compartment between the arbuscules and the PAM, represents a space in which nutrients can be traded between the two organisms. Fueled by a carbon supply from the plant root, the fungus develops extensive extraradical hyphae, outside the root, thereby facilitating the uptake and transfer of mineral nutrients from the surrounding soil [1].

The formation of intracellular fungal structures and the level of fungal proliferation within the roots are dynamically tuned by the plant, which may prevent excessive colonization and carbon loss, thus, maintaining the symbiotic benefits, at mutualistic levels. To achieve this regulation, continual signaling-exchange between the symbionts and an extensive transcriptional reprogramming and cellular remodeling in the plant, are required [10]. Plant hormones are well-known to be key signaling-regulators that act in almost all physiological processes of the plant, including the interactions between the plants and the microbes [11,12,13]. A growing body of evidence has pointed to the crucial role of several plant hormones, such as strigolactones (SLs), gibberellin (GA), and auxin, in the regulation of AM symbiosis. Several reviews regarding the potential roles of phytohormones in AM symbiosis have been published several years ago [14,15,16]. However, as phytohormone-signaling in AM development is an emerging research area, a great number of new findings with respect to the phytohormonal regulation and potential interactions during the establishment of AM symbiosis, have been reported. DELLA proteins, a small group of GRAS transcriptional regulators, have been revealed to be a central node in many signaling pathways, including hormone crosstalk during nodulation and AM colonization. Here, we provide an overview of the recent progress about the roles of these phytohormones and their cross-talks involving the DELLA regulators, in controlling AM development.

## 2. Strigolactones

**Figure 1:**
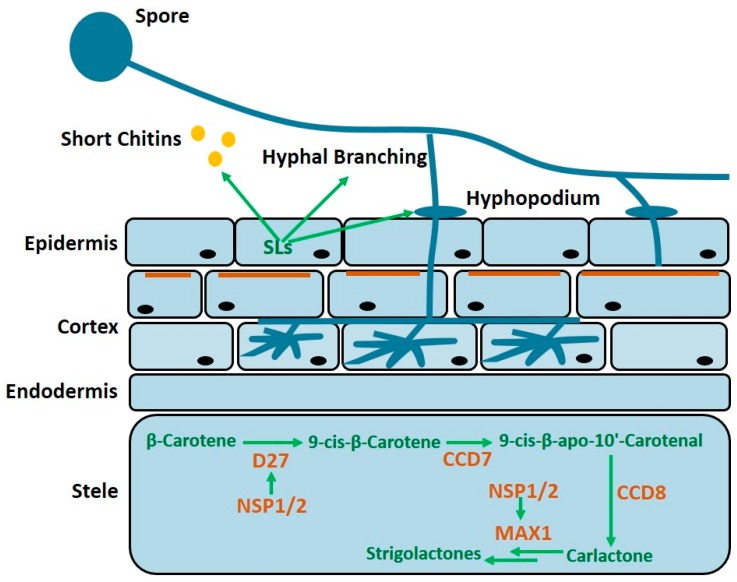
Summary of Strigolactones (SLs) biosynthesis and the effect of SLs on AM development. The biosynthesis of SLs, partially localized in the vasculature of the entire plant, where the D27, CCD7, CCD8, NSP1, NSP2, and MAX1 are all involved in the biosynthesis of SLs [11]. PDR1 protein (brown solid line) is apically localized in the outer cortex [31]. SLs induce the production of a short chitin (CO4, CO5) by the AM fungi, promote hyphal branching [25], and is required for the formation of hyphopoduim [30].

Strigolactones (SLs) are the newly defined phytohormones with roles involved in many aspects of the physiological processes, including seed germination, shoot, and root architecture, as well as AM interaction [17]. Development of AM symbiosis begins with signal communication that occurs prior to the physical contact between plant roots and the AM fungus, and current findings suggest that SLs initiate the molecular dialogues. SLs are a group of carotenoid-derived compounds that are produced in plant roots, and their biosynthesis and exudation are strongly induced, upon a phosphate or nitrogen starvation [18,19,20]. When released into the soil, SLs trigger developmental responses of the AM fungi, such as stimulating spore germination and priming hyphal growth and branching, thereby enhancing the chance of a physical contact with a root [21,22,23,24] (Figure 1). In response to the stimulation, the AM fungus increases the exudation of some chemical molecules, termed “Myc factors”, including chitinoligomers, such as chitin tetramers and pentamers. These fungal-produced signaling molecules are able to elicit pre-symbiosis responses in root tissues, such as induction of nuclear calcium spiking in the rhizodermis and activation of a common symbiosis signaling pathway (CSSP), which are necessary for initiation of the AM symbiosis and also for rhizobial infections, during nodulation [25].

Multiple kinds of SLs, such as strigol, orobanchol, and carlactone, have been isolated from different plant species. The known, naturally occurring SLs consist of a butenolide ring (D ring) linked by an enol ether bridge, to a less conserved second moiety. Genetic studies revealed several proteins, including β-carotene isomerase (DWARF27, D27), two carotenoid cleavage dioxygenases CCD7 and CCD8, and a cytochrome P450 (MAX1), involved in the sequential biosynthesis of SLs [17] (Figure 1). The expression of D27 and MAX1 is controlled by two GRAS transcription factors, NODULATION SIGNALING PATHWAY 1 and 2 (NSP1) and (NSP2), both of which were originally considered to function specifically in nodulation, but are highly conserved in the non-legume plant species. It was recently shown that NSP1 and NSP2 are also required for the AM fungal-associated lipochitooligosaccharide (LCO) signaling, or an AM infection. In *M. truncatula* and rice, *D27* transcripts were barely detectable in roots of *nsp1*, *nsp2*, and *nsp1, nsp2* mutants. Correspondingly, the *d27* and *nsp1/2* double mutant do not produce detectable amounts of SLs [26]. SL levels were also not detectable in root exudates or in root extracts of *ccd7* and *ccd8* mutants, highlighting that the two carotenoid cleavage dioxygenases are also essential for SL biosynthesis [27,28,29]. Even in the absence of a full understanding of how plants release these SLs into the rhizosphere, there is the evidence that export of SLs is associated with an ABC transporter PDR1. In *Petunia hybrida, pdr1* mutants are defective in the SL exudation from their roots, suggesting that PDR1 might function as a cellular SL-exporter [30,31].

The role of SLs serving as the rhizosphere signals, in attracting the AM fungi, have been demonstrated in several plant species, through the analysis of SL-deficient mutants and applications of SL analogs to the rhizosphere. Plant mutants with a defect in SL biosynthesis (*ccd7*, *ccd8*, *nsp1/2*) and export (*pdr1*) showed declined levels of AM colonization and hyphopodium formation, but morphologically normal intraradical fungal structures. Application of the synthetic SL analog GR24 increased the AM colonization in the pea *ccd8* mutant to a similar level as in the pea wild-type with normal strigolactone levels [21,27,28,30,31,32]. These findings highlight the important role of SLs involved in the control of early steps of the AM interaction. However, the colonization defect of *L. Japonicus nsp1* mutant could not be fully restored by the GR24 application, suggesting that NSP1 as the component of CSSP, may perform an additional function in regulating AM formation [33]. It has been recently shown that NSP1 and NSP2 form a hetero-complex that are required for the initiation of nodulation, by associating with promoters of Nod-factor-inducible genes, such as *ENOD11*, *NIN*, and *ERN1* [34,35]. Whether the formation of the NSP1-NSP2 complex is necessary for the initiation of SL biosynthesis remains to be determined.

The perception of SLs in plants is mediated by two proteins, the leucine-rich-repeat F-box protein MAX2/D3/RMS4, and the α/β-fold hydrolase D14/DAD2. MAX2/D3/RMS4 acts as a recognition subunit of the SKP1-CUL1-F-box (SCF) ubiquitin ligase complex, and is thought to target proteins for proteasomal degradation, while D14/DAD2 belongs to a protein family that also includes the gibberellin receptor GID1 [17]. In rice and pea, *d3*/*rms4* mutants showed a similar defective phenotype of the AM colonization—the fungus forms abnormal hyphopodia at the rhizodermis and only very rarely penetrates into the inner cell layers—suggesting that D3-mediated signaling occurs at an early stage of the symbiosis. Interestingly, AM colonization was independent of D14, and the SL-insensitive rice *d14* mutants even showed a higher colonization rate than the wild-type [36,37]. A recent study suggested that the D3-mediated symbiosis control might be associated with the KARRIKIN-signaling by interacting with another α/β-fold hydrolase DWARF14LIKE (D14L). It is worth emphasizing that although SLs are known to play important roles in the initiation of AM symbiosis, SLs may not be the only signal molecules released by plant roots during the pre-contact stage [37,38]. In maize and rice, the no perception 1 (NOPE1) transporter that has the *N*-acetylglucosamine transport activity, is also required for the priming of the fungus. *Nope1* mutants showed almost no interaction with AM fungi and their root exudates failed to trigger transcriptional responses in the fungus. These findings, thus, lead to the hypothesis that the plant-derived *N*-acetylglucosamine-based molecule transport, mediated by the NOPE1 may also function as a key priming signal for the AM fungi, to promote symbiosis [38].

## 3. Gibberellin

Gibberellic acids (GAs) are one of the longest-known classes of phytohormones that can modulate various plant developmental processes, including germination, dormancy breaking, stem elongation, and flowering [13]. It has been repeatedly reported that the AM fungal colonization led to a substantial increase of GA levels in mycorrhizal roots [39,40]. Transcriptome analysis of several mycorrhizal plants also revealed a significantly upregulated expression of multiple genes associated with GA biosynthesis and signaling, upon AM symbiosis. These results led to a suggestion that GAs may also play an important role in the AM development.

GAs have been originally thought to act as a negative factor in AM symbiosis, as several studies, by application of GAs to the mycorrhizal roots, revealed a strong inhibition in the fungal colonization of the host roots [14,40,41]. Consistent with this, the GA-deficient pea mutant, *na-1*, showed a substantially increased AM colonization and arbuscule incidence, which could be reversed by application of GA_3_ [14]. However, a dose-dependent regulation of the AM colonization was observed from the GA-treatment mycorrhizal roots of pea, such that lower GA-concentrations inhibited the formation of arbuscules, while higher concentrations fully suppressed colonization [42]. Not only that, but a positive effect of GA-signaling on the AM development has also been explored in the very recent studies. In *Lotus japonicus*, inhibition of the GA biosynthesis or suppression of the GA signaling repressed the AM-induced subtilisin-like serine protease1 (SbtM1) expression which is required for the AM colonization, and resulted in a significant suppression in hyphal branching and arbuscule formation, in the host root [43]. These results suggest that GA-signaling has a dual role, depending on the endogenous GAs levels, in the regulation of the AM colonization and the arbuscule formation.

The molecular evidence for the action of GA in the AM colonization was also provided by the analysis of GA-response mutants and transgenic plants. DELLA proteins (DELLAs), a small group of putative transcriptional regulators belonging to the plant-specific GRAS family, are predicted to function as key suppressors in the GA-signaling. In the presence of the bioactive GAs, DELLA proteins interact with the GA receptor GID1 (GIBBERELLIN INSENSITIVE DWARF1), and subsequently, are degraded via the 26S proteasome pathway [13]. In rice, the AM colonization level of the GA receptor mutant *gid1* was not affected when treated with GA_3_, however, the wild-type showed a greatly reduced colonization level as compared with the non-treated control. Loss-of-function mutation of the DELLA genes led to a severely reduced incidence of arbuscules in pea, *M. truncatula* and rice (*Oryza sativa*) [14,41,44]. Consistent with this, the overexpression of the unique rice DELLA *SLR1*, resulted in a substantially increased AM colonization, as compared to the wild-type rice plants [41], and the expression of a dominant DELLA protein, non-degradable by GA (D18DELLA1) in the *M. truncatula*, promoted arbuscule formation and counteracted the negative effects of the GA application [40]. These results provide direct evidence that GAs modulate AM colonization via the DELLA proteins, which could, in turn, promote arbuscule formation, through the suppression of GA-signaling. These findings also provide the evidence to support that there exists a precise mechanism in plants to finely tune the GA-signaling and the protein amount of the DELLAs, during the establishment of the AM symbiosis.

**Figure 2:**
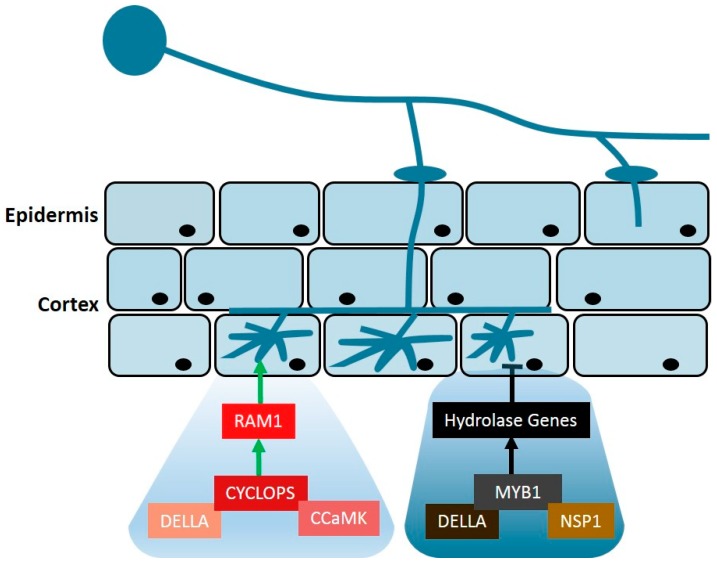
The function of the DELLA proteins in the arbuscule branching and degeneration. In legume, CYCLOPS forms a protein complex by interacting with DELLA and CCaMK, to positively regulate the expression of RAM1 required for the formation of arbuscule [45]. Additionally, MYB1 forms a protein complex by interacting with DELLA and NSP1 to induce the expression of hydrolase genes enhancing the degeneration of arbuscule [48].

DELLA proteins that can interact physically with diverse transcriptional regulators, are known to be involved in many signaling pathways. An increasing amount of evidence has also pointed to DELLAs being a central node, controlling the AM development [40,41,45,46]. In the *M. truncatula*, DELLA1 intersects with a MYCORRHIZA-INDUCED GRAS transcription factor MIG1, to control the cortical radial cell expansion, during the arbuscule development [46]. DELLA proteins can interact with IPD3/CYCLOPS, a component of the CSSP (common symbiosis signaling pathway), to activate the expression of the REDUCED ARBUSCULAR MYCORRHIZA1 (RAM1), a GRAS-domain transcription factor that is required for the arbuscule branching, and could also fine-tune the plant biosynthesis and transfer of lipids to the fungal arbuscules [45,47] (Figure 2). DELLA proteins were also revealed to be required for nodule development and infection-thread formation, during root nodule symbiosis, by promoting the CCaMK-IPD3/CYCLOPS complex formation and bridging a protein complex containing the IPD3/CYCLOPS and NSP2 [34,35]. These findings highlight that DELLA proteins are common components of the symbiotic AM and the rhizobial-signaling pathways. Another interesting finding regarding the DELLAs, during the AM symbiosis, is that they are also associated with the arbuscule premature degeneration in the *mtpt4* mutant. DELLA proteins and the common component NSP1 can physically interact with MYB1, a mycorrhiza-specific MYB-like transcription factor that regulates the expression of a set of the arbuscule degeneration-associated hydrolases, to influence the arbuscule degeneration, during the AM symbiosis [48] (Figure 2).

## 4. Auxin

Auxin was the first identified phytohormone that plays crucial roles in various physiological processes during plant growth and development [12]. The involvement of auxin-signaling in controlling the lateral root initiation and growth, in association with the Pi-signaling, makes this phytohormone a suitable candidate in the AM involvement [49,50,51]. In recent decades, many studies have focused on the role of auxin in regulating AM interactions, and an increased auxin content in mycorrhizal roots, compared with nonmycorrhizal roots, has been recorded for diverse plant species [52,53,54]. Consistent with this, a recent study assayed the expression of DR5-GUS, which is an auxin-responsive reporter construct, and showed a remarkably-increased auxin response in mycorrhizal roots of tomato, *Medicago*, and rice plants, particularly in the arbuscule-containing cortical cells [55].

Further evidence supporting the role of auxin in the AM symbiosis was obtained from mutant studies. AM assessment of the two auxin-related tomato mutants, *dgt*, which is defective in auxin signaling, and *pct*, a mutant with hyperactive polar auxin transport, and an auxin-deficient pea mutant *bsh*, showed a strong reduction in the AM colonization rate, but no defects in the development of fungal structures, leading to a hint that auxin is involved in the AM-initiation, but not in arbuscule differentiation. The *bsh* mutant, which produces three times less auxin in its roots, also showed a significant decrease in the SL exudation and downregulation of a key SL synthesis gene, *PsCCD8*, and its defect in colonization could be partially restored by the application of GR24 [56,57]. These results suggest that the reduced colonization of the *bsh* mutant might be partially ascribed to the low SL synthesis and exudation. It has been documented that auxin could regulate the expression of SL biosynthesis genes, such as *MAX3* and *MAX4* [58]. SLs, in turn, may function as modulators of auxin flux, to control secondary growth, such as shoot branching and lateral root formation, through modulating the localization and expression of auxin transporters, in particular, the auxin efflux PIN transporters [59,60]. Auxin has also been suggested to be involved in the SLs-mediated Pi response in roots, by downregulation of PIN2 to dampen the auxin transport and induction of the TRANSPORTER INHIBITOR RESISTANT 1 (TIR1), to increase the auxin perception [60]. Based on these findings, it is tempting to speculate that auxin-signaling may regulate early events in the formation of the AM symbiosis, in combination with the SL-signaling.

An increasing amount of evidence has suggested that auxin may also be involved in the post-infection stage of mycorrhiza symbiosis [55,61]. Application of low concentrations of synthetic auxin analogs, NAA, and 2,4-D, could stimulate an AM colonization, in particular, arbuscule formation in the mycorrhizal roots of three different plant species—tomato, *Medicago*, and rice [55]. Additionally, the concentrations of free auxin and auxin conjugates was observed to be significantly increased in the mycorrhizal roots of various plant species. In accordance with this, a tomato GH3 (Gretchen Hagen3) gene, *SlGH3.4*, encoding an Indole-3-acetic acid (IAA) amido synthetase, that can inactivate free IAA via the conjugation of IAA to different amino acids, was specifically expressed in mycorrhizal roots, and mainly confined in the arbuscule-containing cells [61,62]. These results highlight the presence of an intricate system that modulates free and conjugated auxin in mycorrhizal plants, to control the development of arbuscules and the maintenance of the symbiosis.

Auxin promotes an interaction between TRANSPORTER INHIBITOR RESISTANT 1/AUXIN SIGNALING F-BOX (TIR1/AFB) and AUXIN/IAA proteins, leading to the degradation of AUXIN/IAA proteins and the release of ARF repression that can then activate the auxin-responsive gene transcription [12]. Overexpression of a mycorrhiza-downregulated microRNA, *miR393*, which targets the auxin receptor, TIR1/AFB, repressed AM colonization, and severely impaired the formation of arbuscules in tomato, *Medicago*, and rice, provides further evidence that arbuscule formation, functioning or degradation is accompanied by an auxin response [54]. Interestingly, a recent study reported that *Sl-IAA27*, a repressor of the auxin-signaling, positively regulates the AM colonization of tomato by regulating the expression of SLs biosynthesis-related genes, *NSP1*, *D27*, and *MAX1* [59]. Knock-down of *Sl-IAA27* had a negative impact on the AM colonization, but did not impair the arbuscule formation. Application of the *Sl-IAA27*-silenced plants with the GR24 could complement their mycorrhizal defect phenotype [59]. These findings suggest that *Sl-IAA27* mediates the early colonization regulation that is dependent on the SL synthesis. The discrepancy in the arbuscule morphology between the miR393-overexpressed plants and the Sl-IAA27-silenced plants suggests that IAA27 is not associated with the miR393-TIR1/AFB-mediated auxin perception signaling pathway, or miR393 might be able to regulate other targets that are required for the arbuscule-branching.

Interactions between the auxin and the GA signalings have also been revealed to play a role in the control of plant growth. Auxin application increases the GA biosynthesis in shoots of the garden pea [58], while application of the auxin biosynthesis inhibitors, downregulated the GA synthesis genes and upregulated the GA deactivation genes, which decreased the bioactive GA level, to stabilize the DELLA protein. In *Arabidopsis*, DELLA could directly interact with ARF to block the DNA-binding activities of the ARF [60]. Therefore, it would be interesting to investigate the roles of the auxin-GA interactions in AM symbiosis.

## 5. Abscisic Acid and Ethylene

Abscisic acid (ABA) is a key abiotic stress signal that modulates many plant physiological processes, such as stress resistance, senescence, and bud dormancy [63,64]. Previous studies in several plant species have revealed an altered ABA level in mycorrhizal plants, compared with nonmycorrhizal plants, but without any consensual conclusion [14]. Genetic evidence for the action of ABA in AM symbiosis was first obtained from the assessment of an ABA biosynthesis-defective tomato mutant, *sitiens* [65]. AM colonization, arbuscule formation, and functionality are impaired in *sitiens* [65], even though a residual amount of the ABA could still be detected in this mutant [66]. These results suggest that ABA may positively regulate the AM development [65,66]. However, as the *sitiens* mutant exhibits an enhanced ethylene (ET) level, the defect in the AM colonization of *sitiens* was suggested to be at least a partially, indirect effect on the ethylene-signaling [65,67].

It has been previously shown that ABA acts as a negative regulator during the root nodule symbiosis, by inhibiting the early signaling at the root epidermis, such as the nodulation factor-induced calcium spiking and early gene expression [68,69]. A recent study reported that ABA may modulate the AM symbiosis, in a concentration-dependent manner, in the *M. truncatula*, which promotes fungal colonization at low-concentrations, and impaired it at high concentrations. High concentrations of ABA seemed to impair the Myc factor-induced (NS-LCO) activation of the symbiotic signaling pathway, while permissive ABA concentrations had no influence on NS-LCO-induced calcium spiking, suggesting the different modes of action of ABA on the AM symbiosis [70]. The positive effect of ABA on the AM colonization, requires a PROTEIN PHOSPHATASE 2A (PP2A) holoenzyme subunit, PP2AB’1, which is induced upon the AM fungal infection and regulated upon the ABA treatment. Mutations in the PP2AB’1 caused a reduction, by 50%, of the AM root-length colonization, however, had no observable effect on the development of fungal structures, including hyphae, arbuscules, and vesicles. The *pp2ab’1* mutants showed no significant difference in both the infection threads and the nodule numbers, as compared to the wild-type, when inoculated with *S. Meliloti*, suggesting that PP2AB’1 is required for an appropriate AM colonization, but not for nodulation, in the *M. truncatula* [70]. Since that ABA treatment was also shown to be able to stabilize the DELLA proteins in the presence of the GA [71], it is tempting to speculate that the ABA-mediated control of the AM development might have direct or indirect connections with the DELLA-mediated signaling pathways.

Ethylene (ET), the gaseous plant hormone, participates in many physiological and developmental processes, from seed germination to fruit ripening. It has previously been shown that ABA-signaling interacts antagonistically with the ET-signaling pathway, and thus ABA and ET are considered to act together in the AM symbiosis. Several studies regarding the assessment of ET-related mutants in tomato and pea, such as the ET-overproducing mutant *epinastic* (*epi*), and the ET-insensitive mutants *rin* (ripening inhibitor) and *ein2*, suggested that ET performs inhibitory roles in the AM development, which is consistent with a role for ET in reducing the symbiotic development, under stressful conditions [72,73]. ET-signaling has also been suggested to be integrated at the level of the DELLA function. An et al. (2012) showed that DELLA could inhibit the transcriptional activity of the ET-stabilized transcription factors EIN3/EIL, by binding to their DNA-binding domains [74]. These results thus give a hint that DELLA proteins may also be involved in the ET signaling-mediated control of the AM symbiosis. However, Foo et al. (2016) revealed an independent effect between the ET, the GA, and the brassinosteroid, on AM development, by an analysis of the double mutants *ein2 na* and *ein2 lk*, that were produced by crosses between *ein2*, the severely GA-deficient *na* and brassinosteroid-deficient *lk* mutants [73]. A recent study of AM assays with the *epi*, *rin*, and NRO ET-responsive mutants, revealed that ET may alleviate the suppressive effect of Pi, on AM formation [75]. In the *epi* mutant which is more sensitive to ethylene, the inhibition of AM formation by a high concentration of Pi, is reduced, as compared to WT.

## 6. Jasmonic Acid, Salicylic Acid, Cytokinins, and Brassinosteroids

Jasmonic acid (JA) is well known for its contribution to the induction of the plant systemic resistance to pathogenic insect attacks [76]. An increasing amount of evidence has suggested that plant systemic resistance could also be induced by interactions with beneficial microbes [77]. The role of JA in the AM colonization has been investigated in several plant species, including *Medicago*, tomato, tobacco, and rice, however, contradicting results have been reported with respect to a neutral, promotive or inhibitory effect, depending partially on the plant species and fungal strains [14,78]. For example, Tejeda-Sartorius et al. (2008) showed that the JA-deficient tomato *spr2* mutant inoculated with *Glomus fasciculatum* had a reduced AM colonization, which could be restored by a methyl jasmonate application [79]. On the other hand, Herrera-Medina et al. (2008) reported an increased AM colonization in the tomato JA-insensitive mutant *jai-1*, when it was inoculated with *Glomus intraradices*, which was supported by a reduced colonization of the wild-type, treated with methyl jasmonate [80]. A recent study of the analysis of a rice mutant *constitutive photomorphogenesis 2* (*cpm2*), that is deficient in JA biosynthesis, suggested that JA is not essential for AM colonization of rice, but high levels of JA in the roots have a suppression effect on the AM development, probably through the induction of defense [81]. JA-signaling has recently been shown to interact with the GA-signaling in mediating the balance between plant growth and the defense against herbivores and pathogens, by controlling the interaction activity between the DELLA and JAZ regulatory proteins, which are involved in the GA and JA signals [80]. The presence of an interaction between the JAZs and the DELLAs leads to a hint that degradation of JAZs by JA may also have an impact on the activity of DELLAs, thus affecting the DELLA-mediated AM development [82].

Salicylic acid (SA) is involved in endogenous signaling that induces a systemic acquired resistance (SAR) to pathogens, and is expected to be activated during the AM symbiosis, which also implicates the fungal hyphae, invading the plant cells. Even so, much less is known about the roles of the SA in the AM symbiosis, thus far. An exogenous application of the SA to rice roots, decreased the root colonization at the onset of the symbiotic interaction, but showed no influence on appressoria formation, suggesting that the SA may not have a direct inhibitory effect of on the fungal growth [83]. Blilou et al. (1999) showed that in the AM-defective (Myc^−^) mutants of *P. sativum*, the SA accumulation was increased [84]. Herrera-Medina et al. (2003) reported that the transgenic NahG plants that possessed reduced levels of SA had more rapid AM colonization, while the transgenic CSA plants with constitutive SA biosynthesis exhibited retarded-AM colonization, although the final level of colonization was not significantly altered [85]. These results suggest that alteration of the plant endogenous SA content may increase or delay the AM colonization process, but did not affect the maximal degree of root colonization.

Cytokinin (CK) is a classic plant hormone with roles involved in promoting cell division and organ formation [86]. Although CK has been revealed to play essential roles in nodule formation, the regulatory role of CK in AM colonization is not well understood [87]. An increased accumulation of CK in both shoots and roots have been documented by early studies for several AM plants [88,89,90]. By analysis of a CK-insensitive *M. truncutula cre1* mutant, Plet et al. (2011) suggested that CK may not be essential for the regulation of AM development [91]. Cosme et al. (2012), however, showed that reducing CK levels in tobacco by a constitutive expression of CK-degrading *CKX2* gene (35S:CKX2) could stimulate AMF (*Rhizophagus intraradices*) hyphal growth in the roots, resulting in higher percentages of AMF root colonization than that of the WT [88]. A more recent study using transgenic tobacco plants, with a root-specific or constitutive expression of the CK-degrading *CKX* genes, suggested that shoot CK has a positive impact on the AM fungal development in roots and on the transcription of an AM-specific Pi transporter gene (*NtPT4*). A reduced CK content in roots caused a depression in the shoot and the root growth, following the AM colonization, but had no significant effect on the uptake of P and N, and the expression of *NtPT4* [89]. The authors, thus, gave a proposal that root CK may restrict the C-availability from the roots to the fungus, thus, to avoid parasitism by the AM fungi [89]. Jones et al. (2015) reported a defective phenotype with lower nodule number, enhanced mycorrhizal colonization, and delayed lateral root emergence, in regard to a pleiotropic pea mutant—E151 (*sym15*)—with high root CK levels [90]. Through reciprocal grafts, it was shown that in E151 (*sym15*), the hyphopodium number was regulated by both the root and the shoot, whereas, the numbers of arbuscules and vesicles were controlled by the shoot only, suggesting that CK may play an essential role in promoting the entry of the fungus into the cortex. The other evidence for the action of CK in the AM development could be indirectly speculated from the well-established interactions between the CK and auxin-signalings, in modulating multiple plant physiological processes [92,93]. SHY2/IAA3, a member of the Aux/IAA family encoding auxin-response inhibitors, has been proposed to be part of a feedback control that converges CK and auxin signals to lessen the abundance of several auxin efflux carrier PIN proteins, and to reduce CK biosynthesis by downregulating genes encoding isopentyl diphosphate transferase (IPT) [92,93].

Plant hormone Brassinosteroids (BRs) are critical for plant growth and development, and could promote stem elongation and cell division [94]. To date, very little is known about the effects of BRs on AM symbiosis. The BR-deficient pea mutant *lkb*, which results from a leaky mutation in the gene, which is involved in campesterol production during BR biosynthesis, showed no alteration in the AM colonization, as compared with the wild-type plants [73]. However, the tomato *dX* mutants that are defective in the BR biosynthesis, exhibited a decrease in, both, the AM colonization level and the sugar content [87]. A sucrose transporter, SISUT2, could physically interact with the BR biosynthesis and signaling components to regulate AM symbiosis [95]. In rice, BRs could promote a GA accumulation, by activating the expression of GA metabolic genes [96]. BRs were also shown to be the master regulators of GA biosynthesis in *Arabidopsis*. GA levels were significantly decreased in the BRs biosynthetic *cpd* mutants and BRs-signaling *bri1* mutants [97]. Moreover, BZR1, the key transcriptional factor in the BRs-signaling pathway, could directly interact with DELLAs and negatively control each other’s transcriptional activity. Therefore, a role of the BR-GA interactions in regulating AM symbiosis could be imaged.

## 7. Conclusions and Remarks

The control of AM symbiosis is a finely-tuned process that involves multiple regulatory components functioning at multiple levels. Phytohormonal regulation of the AM development is an emerging area of research and is drawing more and more attention. Research over the past few years, has revealed the critical roles of some phytohormones in modulating AM interactions, from early recognition/colonization events up to the final arbuscular formation and degradation, by analyzing mutants and transgenic plants. However, as the ubiquitous interactions among different phytohormones, and even the AM fungal-produced hormones, become known, we currently still lack deep insight into the molecular mechanisms underlying how plants coordinate different hormones to enable the development of AM symbiosis and avoid parasitism by AM fungi [89]. The increasing use of genetic resources affected in biosynthesis, degradation, or perception of phytohormones in different dicot and monocot plants, coupled with physiological experiments and reporter assays, will be an effective approach to close this gap. Moreover, due to the lack of AM fungal mutants, some old, seemingly simple questions are still hard to get precise answers. For example, enhanced accumulation of auxin in AM roots have been shown for multiple plant species, however, no direct evidence could be traced regarding the origin (from plants or AM fungi, or both) of the accumulated auxin in the colonized cells [55,98]. Therefore, to develop an effective gene-editing tool, for the AM fungi, would be greatly important for dissecting the roles of the phytohormone-signaling in the AM symbiosis.

In the future, accurate and real-time measurement of hormone distributions and concentrations will resolve contradictory findings on AM responses, following the external hormone treatment. DELLA proteins have been revealed to be central regulators involved in many signaling pathways. In legumes, DELLAs function as the key components in the CSSP, required for both root nodulation and AM symbiosis. In the presumed non-host model plant, *Arabidopsis thaliana*, DELLA has been reported to directly or indirectly interplay with almost all the phytohormonal-signalings. Since rudimentary AM phenotypes have been identified in multiple presumed non-host plant species, including the *Arabidopsis*, an exciting future task will be to uncover the evolutionary conservation and divergence of the DELLA-mediated hormonal signaling interactions between the mycorrhizal host and the presumed non-host plant species. Moreover, given the central position of DELLAs in the hormone-crosstalk, in nodulation, further studies on high-throughput screening of DELLA-binding transcription regulators, involved in both mycorrhizal and hormonal-signaling pathways, would open up new perspectives in depicting a bigger picture regarding the symbiosis-mediated hormonal signaling regulatory networks.
